# Garlic oil supplementation blocks inflammatory pyroptosis-related acute lung injury by suppressing the NF-κB/NLRP3 signaling pathway via H_2_S generation

**DOI:** 10.18632/aging.205721

**Published:** 2024-04-12

**Authors:** Tursunay Dilxat, Qiang Shi, Xiaofan Chen, Xuxin Liu

**Affiliations:** 1 Xinjiang Agricultural Vocational Technological College, Changji 831100, Xinjiang, China

**Keywords:** acute lung injury, garlic oil, lipopolysaccharide, anti-inflammatory

## Abstract

Acute lung injury (ALI) is a major cause of acute respiratory failure with a high morbidity and mortality rate, and effective therapeutic strategies for ALI remain limited. Inflammatory response is considered crucial for the pathogenesis of ALI. Garlic, a globally used cooking spice, reportedly exhibits excellent anti-inflammatory bioactivity. However, protective effects of garlic against ALI have never been reported. This study aimed to investigate the protective effects of garlic oil (GO) supplementation on lipopolysaccharide (LPS)-induced ALI models. Hematoxylin and eosin staining, pathology scores, lung myeloperoxidase (MPO) activity measurement, lung wet/dry (W/D) ratio detection, and bronchoalveolar lavage fluid (BALF) analysis were performed to investigate ALI histopathology. Real-time polymerase chain reaction, western blotting, and enzyme-linked immunosorbent assay were conducted to evaluate the expression levels of inflammatory factors, nuclear factor-κB (NF-κB), NLRP3, pyroptosis-related proteins, and H_2_S-producing enzymes. GO attenuated LPS-induced pulmonary pathological changes, lung W/D ratio, MPO activity, and inflammatory cytokines in the lungs and BALF. Additionally, GO suppressed LPS-induced NF-κB activation, NLRP3 inflammasome expression, and inflammatory-related pyroptosis. Mechanistically, GO promoted increased H_2_S production in lung tissues by enhancing the conversion of GO-rich polysulfide compounds or by increasing the expression of H_2_S-producing enzymes *in vivo*. Inhibition of endogenous or exogenous H_2_S production reversed the protective effects of GO on ALI and eliminated the inhibitory effects of GO on NF-κB, NLRP3, and pyroptotic signaling pathways. Overall, these findings indicate that GO has a critical anti-inflammatory effect and protects against LPS-induced ALI by suppressing the NF-κB/NLRP3 signaling pathway via H_2_S generation.

## INTRODUCTION

Acute lung injury (ALI) is a noncardiogenic acute progressive hypoxic lung disease frequently accompanied by diffuse alveolar injury and acute respiratory failure [1, 2]. Its pathological characteristics include alveolar epithelium injury, increased pulmonary microvessel permeability, exudation, pulmonary edema, and substantial inflammatory cell infiltration [3]. This disease affects millions of people globally and has a high mortality and morbidity rate [1, 4]. Lipopolysaccharide (LPS), one of the most important pathogenic antigens of gram-negative bacteria, is typically used to establish animal models of ALI to investigate more effective ways to overcome ALI [5, 6]. Despite tremendous progress in understanding the causative mechanisms of the disease, effective therapeutic strategies for patients with ALI remain limited. Therefore, there is an urgent need to explore more methods and drugs to treat ALI.

Although the pathogenic mechanism of ALI remains unclear, the inflammatory response is one of the key mechanisms that has been conclusively shown to cause ALI [7]. In a previous study on a mouse model of ALI, the levels of tumor necrosis factor alpha (TNF-α) and interleukin (IL-1β), as representatives of many inflammatory cytokines, were found to be elevated in bronchoalveolar lavage fluid (BALF) [8]. Additionally, another study reported that LPS-induced ALI was alleviated through the suppression of the expression of numerous inflammatory cytokines [9, 10]. Studies have confirmed that nuclear factor-κB (NF-κB) signaling is a key pathway that regulates the production of these inflammatory cytokines. Suppression of NF-κB signaling attenuated LPS-induced alveolar epithelial cell injury and LPS-induced ALI [11, 12]. The NLRP3 inflammasome that mediates IL-1β and IL-18 maturation also plays a vital role in ALI progression [13]. Pyroptosis is a new type of inflammatory programmed cell death that is activated by NLRP3 and causes the release of multiple gasdermin family members, including gasdermin E (GSDME) and gasdermin D (GSDMD) [14, 15]. Gasdermins, considered as indicators of pyroptosis, are responsible for cell perforation. Pyroptosis also plays a critical role in promoting unfavorable developmental outcomes in ALI inflammation [16, 17]. Consequently, the development of anti-inflammatory and antipyroptotic drugs for treating ALI is preferred.

In the past few years, the role of nutrition in disease prevention and treatment has received rapid attention. Studies have revealed that many active ingredients derived from food can prevent or treat ALI [18, 19]. However, the availability of some ingredients from their natural sources is highly limited. Recently, accumulating evidence has indicated that garlic, a globally used cooking spice, has a myriad of beneficial effects [20, 21]. Garlic exhibits excellent anti-inflammatory bioactivity [22, 23] and inhibits NF-κB activation [24]. Owing to the relatively low cost, remarkable effectiveness, and few side effects, the consumption of garlic has progressively increased worldwide [25]. Additionally, leveraging the therapeutic potential of its bioactive molecules and discovering the underlying mechanism is crucial for reducing healthcare costs and supporting economic development in rural communities.

Research suggests that the large amount of organosulfur compounds that garlic contains, such as diallyl trisulfide (DATS) and diallyl disulfide, is the key to its medicinal value [26, 27]. Ko et al. revealed that garlic oil and diallyl disulfide could prevent cigarette smoke-induced airway inflammation in mice, confirming their therapeutic potential in pulmonary diseases.

H_2_S released from these organic polysulfides under physiological conditions is a key biomaterial in the regulation of physiological and pathological diseases, which can exert protective effects against ALI through various pathways, such as antioxidant stress, antinitrification stress, anti-inflammation, and anti-ferroptosis [28, 29]. Additionally, GO supplementation has been reported to upregulate the levels of H_2_S-producing enzymes, including cystathionine β-synthase (CBS), cystathionine γ-lyase (CSE), and 3-mercaptopyruvate sulphurtransferase (3MST) [30–32]. H_2_S is involved in respiratory activities and can affect the outcomes of various lung diseases [33]. A previous study revealed that serum levels of H_2_S in patients with chronic obstructive pulmonary disease are lower than those in healthy individuals [34]. Moreover, H_2_S can exert an antioxidation effect by attenuating the levels of glutathione disulfide in the lungs of hypoxia-induced pulmonary hypertensive rats. Furthermore, Wang et al. revealed that H_2_S exerts its antifibrotic activity by suppressing the TGFβ/Smad pathway [35]. Notably, H_2_S alleviates acute kidney damage by suppressing NLRP3 activation and pyroptosis [36]. Because NLRP3 plays a critical role in ALI inflammation, we speculated whether GO exerts it protective effect against ALI by inhibiting the NF-κB/NLRP3 signaling pathway via H_2_S generation.

Therefore, the present study aimed to investigate the therapeutic effect of GO on LPS-induced ALI and its intrinsic molecular mechanisms in an ALI mouse model.

## MATERIALS AND METHODS

### Mice protocols

Male C57BL/6 mice (6–8 weeks old) were purchased from Karamay Zhonghui Trading Co., Ltd., (Karamay City, China). All trial procedures were approved by The Ethics Committee of Xinjiang Agricultural Vocational Technological College. Mice were randomized into three groups: control, LPS, and LPS + GO (GO, 100 mg/kg); or five groups: control, LPS, LPS + GO (GO, 100 mg/kg) [37, 38], LPS + GO (GO, 100 mg/kg) + iodoacetamide (IAM); LPS + GO (GO, 100 mg/kg) + aminooxyacetic acid ((AOAA), 50 mg/kg) + DL-propargylglycine ((PAG), 100 mg/kg). For ALI model establishment, the mice were anesthetized via intraperitoneal injection of sodium pentobarbital (50 mg/kg) and then intratracheally injected with LPS (5 mg/kg) dissolved in 50 μl of phosphate-buffered saline (PBS). An equal volume of PBS was applied to control mice as a control. For GO delivery, mice were intratracheally injected with GO (100 mg/kg, dissolved in 50 μl of dimethyl sulfoxide (DMSO)) 4 h before LPS instillation. DATS is an active constituent isolated from GO, and IAM is an effective inhibitor of the conversion of DATS to H_2_S *in vivo*. For IAM delivery, mice were administered 3% IAM in their drinking water 30 min after LPS instillation. CBS and CSE are the two main enzymes that mediate H_2_S production *in vivo*. AOAA and PAG are CBS and CSE inhibitors, respectively. For AOAA and PAG delivery, mice were intraperitoneally injected with AOAA (50 mg/kg) and PAG (100 mg/kg) 30 min after LPS instillation. Samples were collected 24 h after LPS instillation. Enzyme-linked immunosorbent assay (ELISA), histological analysis, total protein assays, and cell counting were performed with collected BALF samples. ELISA, immunohistochemistry, real-time polymerase chain reaction (PCR), and western blotting were conducted with collected lung tissues.

### Drugs and reagents

The following compounds were used in this study: LPS (cat. L2880, Sigma-Aldrich, USA), GO (cat. W250309, Sigma-Aldrich), IAM (cat. A3221, Sigma-Aldrich), AOAA (cat. C13408, Sigma-Aldrich), and PAG (cat. P7888, Sigma-Aldrich). Antibodies used were IL-6 antibody (cat. 21865-1-AP, Proteintech, China), TNF-α antibody (cat. 60291-1-Ig, Proteintech), IL-1β antibody (cat. 16806-1-AP, Proteintech), IL-18 antibody (cat. 10663-1-AP, Proteintech), p-NFκB p65 antibody (cat. 3033, Cell Signaling Technology, USA), NF-κB p65 antibody (cat. 8242T, Cell Signaling Technology), NLRP3 antibody (cat. 15101S, cat. 8242T, Cell Signaling), ASC antibody (cat. sc-514414, Santa Cruz Biotechnology, USA), cleaved caspase-1 antibody (cat. sc56036, Santa Cruz Biotechnology), GSDMD antibody (cat. A18281, Abclonal, USA), cleaved N-terminal GSDME antibody (cat. ab222407, Abcam, UK), CBS antibody (cat. GTX628777, GeneTex, USA), CSE antibody (cat. 12217-1-AP, Proteintech), 3MST antibody (cat. HPA001240, Sigma-Aldrich), and β-actin antibody (60004-1-Ig, Proteintech).

### Hematoxylin and eosin (H&E) staining and pathology scores

The lungs were fixed with a 12% neutral formaldehyde solution for 24 h and then dehydrated in a series of graded concentrations of alcohol dilutions. The tissue was then embedded in paraffin, and the paraffin blocks were cut into 4-μm-thick sections. Sections were stained with H&E and then observed under a light microscope for pathological changes in the lung tissues. The ALI score was independently calculated by two pathologists based on parameters including alveolar septal thickening, pulmonary hemorrhage, hyaline membrane formation, and substantial neutrophil infiltration into alveolar spaces [39]. The scoring scale was defined as follows: virtually no damage, 0–1 point; mild-to-moderate damage, 2–5 points; and severe damage, 5–10 points.

### Detection of lung wet/dry (W/D) ratio

The upper lobe of the left lung was dissected out, immediately measured, and used as the wet weight of the lung. The lung tissue was then dried at 65°C for 72 h, and the weight of the dried lungs was measured immediately. The W/D ratio of the lungs was calculated as an indication of the degree of pulmonary edema.

### Lung myeloperoxidase (MPO) activity measurement

Lung tissues used for MPO experiments must be irrigated with PBS to remove all blood. Lung MPO activity was determined using an MPO colorimetric activity assay kit (cat. ab105136, Abcam). The optical density value of MPO was measured using a microplate reader (FilterMax F5, Molecular Devices, USA) at 460 nm.

### Bronchoalveolar lavage

After being euthanized, the chest cavity of the mice was exposed and the trachea was intubated. An 18G sterile and blunt-ended needle was inserted through the resection port into the trachea. A total volume of approximately 2.4 ml of BALF was recovered from each mouse through injection of 2 ml of cold saline. The supernatants were collected via centrifugation and stored. The total protein level of BALF was measured using a BCA protein assay kit (cat. CW0014S, CWBIO, Jiangsu, China). The precipitated cells were resuspended, and the number of cells in BALF was calculated using a grid blood cell counter.

### RNA extraction and real-time PCR

Total cellular RNA was extracted and reverse transcribed. The cDNA was then subjected to a real-time PCR program running on an ABI7500 Real-Time PCR System (Applied Biosystems, USA) with the following thermocycling conditions: initial predenaturation for 2 min at 50°C and 10 min at 95°C, followed by a 40-cycle two-step PCR (95°C for 15 s and 60°C for 1 min). GAPDH was used as an internal reference control. The primer sequences for the RNAs to be detected are listed in Table 1.

**Table 1:** Sequences of PCR primers used in this study.

GAPDH	Forward (5'–3')	AGGTCGGTGTGAACGGATTTG
	Reverse (5'–3')	GGGGTCGTTGATGGCAACA
IL-6	Forward (5'–3')	GGCGGATCGGATGTTGTGAT
	Reverse (5'–3')	GGACCCCAGACAATCGGTTG
TNF-α	Forward (5'–3')	ACCCTCACACTCACAAACCA
	Reverse (5'–3')	ACCCTGAGCCATAATCCCCT
IL-1β	Forward (5'–3')	ATGATGGCTTATTACAGTGGCAA
	Reverse (5'–3')	GTCGGAGATTCGTAGCTGGA
IL-18	Forward (5'–3')	GGCCGACTTCACTGTACAACC
	Reverse (5'–3')	TCTGGGGTTCACTGGCACTTTG
NLRP3	Forward (5'–3')	GCTGCGATCAACAGGCGAGA
	Reverse (5'–3')	AAGGCTGTCCTCCTGGCATAC
ASC	Forward (5'–3')	CTGACGGATGAGCAGTACCA
	Reverse (5'–3')	CAGGATGATTTGGTGGGATT
Caspase-1	Forward (5'–3')	ACACGTCTTGCCCTCATTATCT
	Reverse (5'–3')	ATAACCTTGGGCTTGTCTTTCA
GSDMD-N	Forward (5'–3')	GGAGGAATTAATTGAGGCGGC
	Reverse (5'–3')	GGCACCAGTTCTCCAGAGTC
GSDME-N	Forward (5'–3')	CGTAGAGAGCCAGTCTTCATTT
	Reverse (5'–3')	GTTCCAGGACCATGAGTAGTTC

### Western blot

Lung tissue lysate was prepared with RIPA buffer (cat. P0013B, Beyotime, Shanghai, China) using a tissue homogenizer. Proteins were precipitated via centrifugation, followed by denaturation at 95°C for 10 min. After quantification using a BCA protein concentration quantification kit (cat. CW0014S, CWBIO), proteins of different molecular weight sizes were separated via SDS-PAGE and electrotransferred onto polyvinylidene difluoride membranes (cat. ISEQ00010, Millipore, USA). The membranes were sequentially incubated with various primary antibodies and horseradish peroxidase-labeled secondary antibodies and finally subjected to chemiluminescent color development. The grayscale values of the bands of different sizes of proteins were analyzed using ImageJ software.

### ELISA

The activities of inflammatory factors, including TNF-α, IL-6, IL-18, and IL-1β, in BALF and lung tissues were detected via ELISA using the following kits according to the manufacturer’s instructions: mouse IL-6 ELISA kits (Abcam, ab222503), mouse TNF-α ELISA kit (Abcam, ab208348), mouse IL-1β ELISA kits (Abcam, ab197742), and mouse IL-18 ELISA kit (Abcam, ab216165).

### Isolation of primary alveolar epithelial cells

Primary alveolar epithelial cells were isolated from lung tissues with different treatments using a previously described protocol [40]. Briefly, sterile PBS was instilled into the lungs of mice to drain all the remaining blood. The lungs were then digested with elastase (14 units/ml) and incubated at 37°C for 50 min. After incubation, the pulverized lung tissue was passed through a 20-μm filter to prepare a dispersed single-cell suspension. Fibroblasts and macrophages were removed by adhering them to a Petri dish coated with IgG. Isolated primary alveolar epithelial cells were confirmed via western blotting and immunofluorescence using antibodies targeting their cell-specific SP-C markers.

### Flow cytometry

Caspase-1 activity, an indicator of pyroptosis, was measured using the FLICA 660 Caspase-1 Assay kit (cat. 9122, ImmunoChemistry, USA). First, fetal bovine serum (FBS)-washed and trypsin-digested cells were resuspended in PBS with 2% FBS. Then, 660-YVAD-FMK was added to the cells as a fluorescent inhibitor probe that can label the active caspase-1 enzyme. The proportion of pyroptotic cells was measured using flow cytometry according to the manufacturer’s instructions. BD FACSAria™ II cell sorter (BD Biosciences, USA) was used to analyze the data.

### Detection of H_2_S levels in lung tissues

Lung tissue homogenates were prepared in 100 mM of cold potassium phosphate buffer supplemented with protease inhibitors. Then, 100 μl of homogenate and 1.2 M HCl containing 200 μl of 30 mM ferric chloride (FeCl_3_) and 7.2 M HCl (containing 100 μl of 1% zinc acetate, 100 μl of borate buffer (pH = 10), and 200 μl of 20 mM N,N-dimethyl-p-phenylenediamine dihydrochloride) was mixed and incubated in the dark at 37°C for 15 min. Next, the sample was centrifuged at 4°C and 10000 rpm for 5 min. Subsequently, the supernatants were collected and the OD value was measured at 670 nm using a microplate reader (FilterMax F5, Molecular Devices). Finally, the H_2_S concentration was calculated according to the standard curve of sodium bisulfite solution [36].

### Statistical analysis

All data are presented as the means ± standard deviation. Results were analyzed using GraphPad Prism 8 software. Parameters were compared between two groups using a two-tailed Student’s *t*-test. Differences at *p*-values of < 0.05 were considered statistically significant.

## RESULTS

### GO attenuates LPS-induced ALI

**Figure 1:**
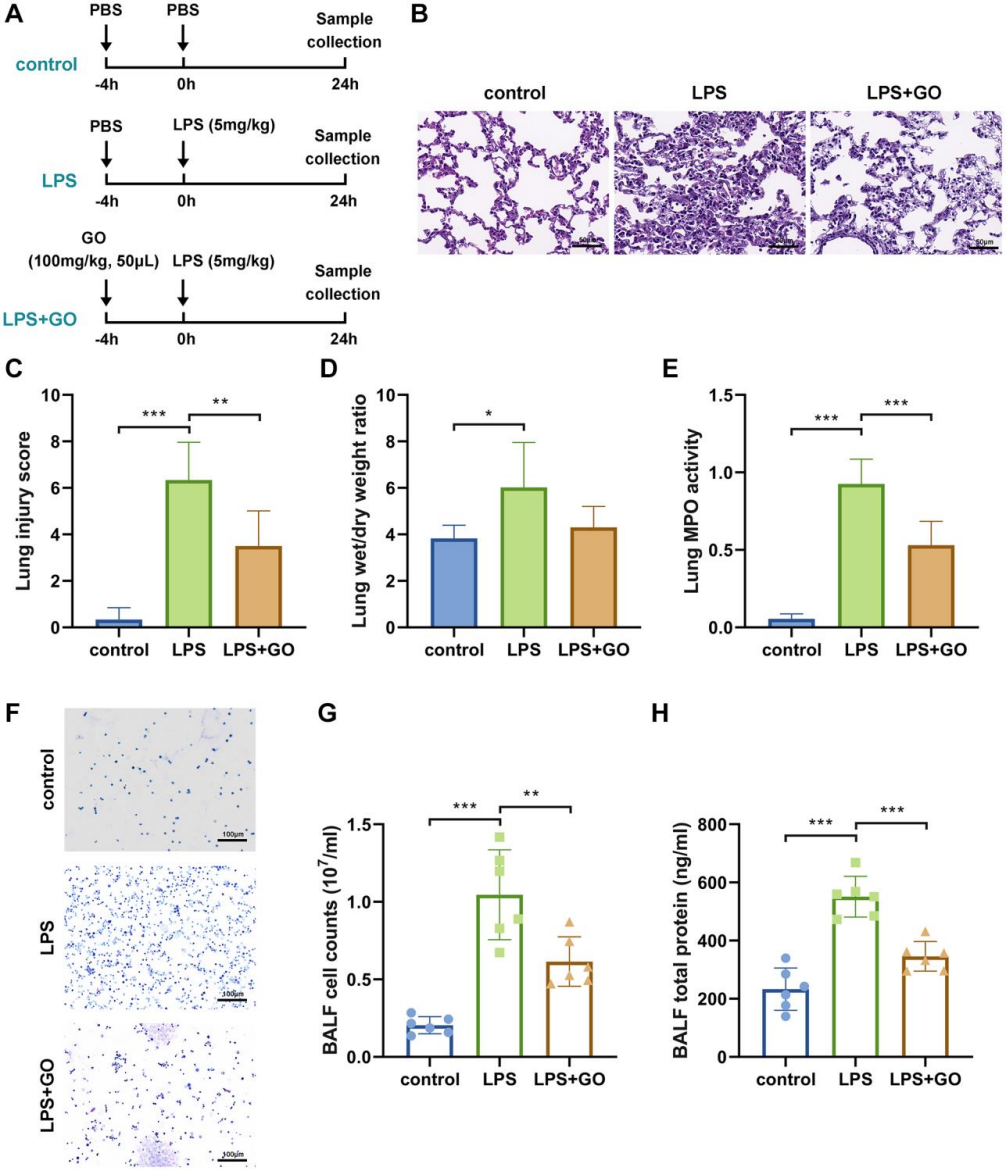
**GO attenuates LPS-induced ALI.** For ALI model establishment, mice were anesthetized via intraperitoneal injection of pentobarbital sodium (50 mg/kg) and then injected intratracheally with LPS (5 mg/kg) dissolved in 50 μl PBS. Control mice were injected with the same volume of PBS. For GO delivery, mice were injected intratracheally with GO (100 mg/kg, dissolved in 50 μl of DMSO) 4 h before LPS instillation. Samples (lung tissues and BALF) were collected 24 h after LPS instillation. (**A**) Flow chart of mouse experimental design. (**B**) Lung tissue sections stained with hematoxylin and eosin (H&E staining, scale bar: 50 μm). (**C**) Severity of lung damage was scored on a scale of 0–10 according to inflammatory cell infiltration, alveolar septal edema and thickening, hyaline membrane formation, and pulmonary hemorrhage (*n* = 8 per group). (**D**) Effect of GO on lung wet/dry ratio. (**E**) Quantification of MPO activity in lung tissue. (**F**) Cells were precipitated via BALF centrifugation and stained with Wright-Giemsa. (**G**) Cell counts in BALF samples from mice subjected to different treatments. (**H**) Total protein measurement in BALF via BCA assay. Experiments were performed in triplicate, and data are expressed as the mean ± standard deviation (SD). Student’s *t*-test; ^*^p < 0.05, ^**^p < 0.01, and ^***^p < 0.001 defined. Abbreviation: ns: no significant difference.

To investigate the effect of GO on ALI, we used a mouse model of LPS-induced ALI and administered GO at a dose of 100 mg/kg (Figure 1A). Histopathological features and scores indicative of the extent of lung injury in the LPS-induced ALI model are shown in Figure 1B, 1C. Compared to control mice, the lung tissue receiving LPS injections showed significant pathological changes, including interstitial lung edema, substantial infiltration of inflammatory cells, and hemorrhage in intra-alveolar and thickened interalveolar septa, indicating the presence of ALI. However, 100 mg/kg of GO pretreatment improved lung structure and inflammatory cell infiltration and significantly reduced lung injury scores (Figure 1B, 1C). However, no inhibitory effect of GO pretreatment on LPS-induced elevation of lung W/D ratio was observed (Figure 1D). Additionally, GO pretreatment remarkably reduced the level of MPO, an indicator of neutrophil infiltration, in the lungs, which was substantially elevated in LPS-induced ALI (Figure 1E). These results indicate that GO has a protective effect on LPS-induced lung injury.

BALF is also an important indicator of the severity of LPS-induced ALI and whether or not GO is protective. The degree of inflammatory cell infiltration into the alveoli was measured by staining cells collected from BALF. LPS significantly increased the number of cells in BALF, whereas GO pretreatment remarkably reduced the number of cells (Figure 1F, 1G). Furthermore, the total protein level inside BALF, as an indicator of pulmonary edema occurrence, can be used to evaluate pulmonary capillary permeability. As expected, compared with the control, the total protein level in BALF was much higher in the LPS group. However, GO pretreatment reversed the LPS-induced increase in the total protein level in BALF, indicating that GO prevented the LPS-induced increase in capillary permeability (Figure 1H).

### GO blocks lung inflammation during LPS-induced ALI

**Figure 2:**
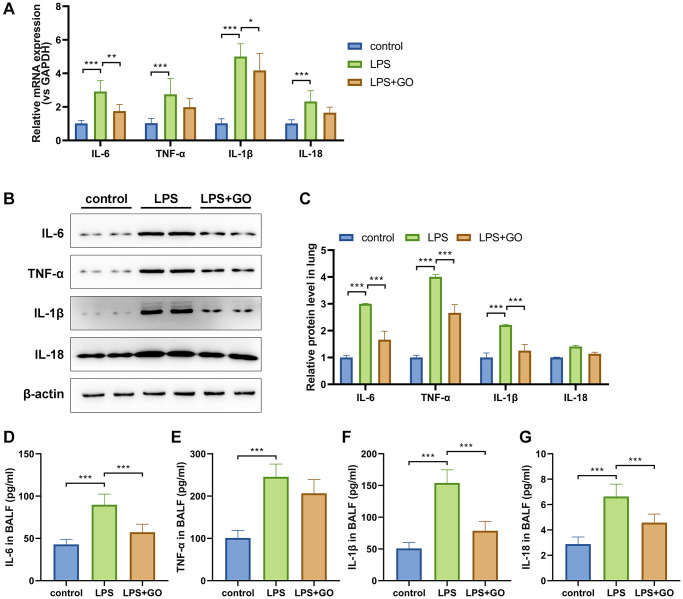
**GO blocks lung inflammation during LPS-induced ALI.** (**A**) Real-time PCR analysis showed that GO prevented the increase in the mRNA expression levels of inflammatory cytokines, including TNF-α, IL-6, IL-1β, and IL-18, in lung tissues. (**B**) Western blotting revealed that GO blocked the increase in the protein expression levels of inflammatory cytokines, including TNF-α, IL-6, IL-1β, and IL-18, in lung tissues. (**C**) Statistical chart of density analysis using ImageJ. (**D**–**G**) ELISA analysis revealed that GO inhibited the secretion of inflammation cytokines, including TNF-α, IL-6, IL-1β, and IL-18, in BALF. Experiments were performed in triplicate, and data are expressed as the mean ± standard deviation (SD). Student’s *t*-test, ^*^p < 0.05, ^**^p < 0.01, and ^***^p < 0.001. Abbreviation: ns: no significant difference.

Dysregulation of the systemic inflammatory response during infection is an important feature of ALI development [41]. The levels of inflammatory factors TNF-α, IL-6, IL-1βand IL-18 in ALI-infected lungs were detected via real-time PCR (Figure 2A) and western blotting (Figure 2B, 2C). The mRNA and protein levels of the aforementioned inflammatory cytokines demonstrated significant upregulation in the lungs that developed ALI after LPS treatment but were partially reduced by GO pretreatment (Figure 2A–2C). BALF was collected to measure systemic inflammation through ELISA, and the results showed that the levels of the aforementioned inflammatory cytokines in the BALF of ALI mice were also remarkably reduced by GO pretreatment (Figure 2D–2G). The findings reveal the vital anti-inflammatory effect of GO on ALI.

### GO inhibits the activation of NF-κB p65 and NLRP3-dependent inflammasome in LPS-induced ALI

**Figure 3:**
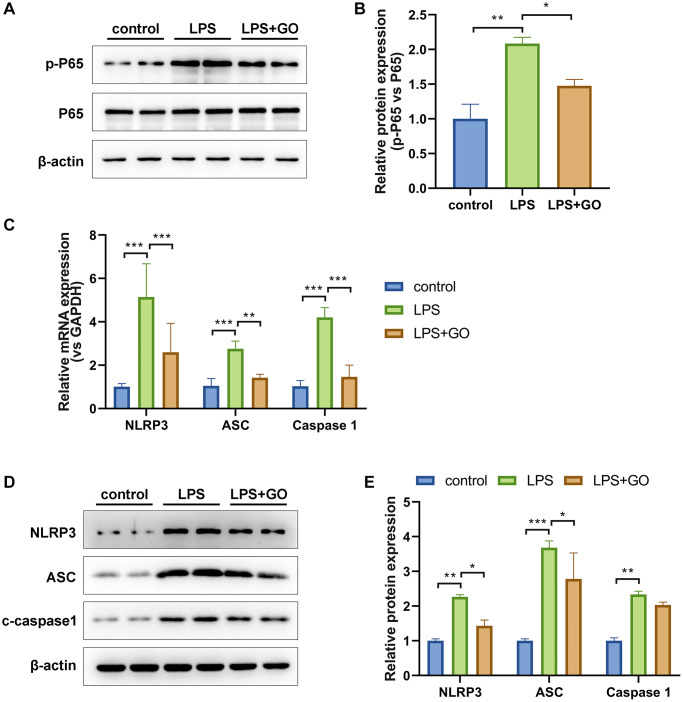
**GO inhibits the activation of NF-κB p65 and NLRP3 inflammasome in LPS-induced ALI.** (**A**) Western blot analysis of phosphorylated NF-κB p65 levels in lung tissue. (**B**) The grayscale values of the bands in image A were analyzed using ImageJ, and the ratios of p-p65/p65 were calculated. (**C**) Real-time PCR analysis of mRNA expression levels of the NLRP3/ASC/caspase 1 inflammasome complex in lung tissue. (**D**) Western blotting analysis of protein expression levels of the NLRP3/ASC/caspase 1 inflammasome complex in lung tissue. (**E**) A histogram of the grayscale values of the protein bands calculated using ImageJ software (standardized to β-actin). Experiments were performed in triplicate. Student’s *t*-test, ^*^p < 0.05, ^**^p < 0.01, and ^***^p < 0.001. Abbreviation: ns: no significant difference.

The production of mature forms of IL-1β and IL-18 depends on NLRP3 inflammasome activation, which is potentially responsible for ALI development [42]. The marked activation of the NF-κB signaling pathway, which is involved in the transcriptional regulation of NLRP3 inflammasome activation and in the production of IL-6, TNF-α, pro-IL-1β, and pro-IL-18, is also closely related to LPS-induced ALI [43]. To further elucidate the mechanisms underlying the suppressive effects of GO on inflammatory cytokines, such as TNF-α, IL-6, IL-1β, and IL-18, in lungs with ALI, we further investigated the effect of GO on the expression of NF-κB and NLRP3 inflammasome pathway-related proteins. The western blotting results revealed significantly elevated protein expression levels of p-p65/p65 in the ALI group compared with the control group. However, GO pretreatment markedly reduced the expression of p-p65/p65 (Figure 3A, 3B). Additionally, we found that gene and protein expression levels of NLRP3, ASC, and cleaved caspase-1, which are the components that make up the inflammatory complex, were elevated in ALI lungs compared with controls and that GO pretreatment also reduced this expression (Figure 3C–3E). These results suggest that GO prevents endotoxemia-associated lung inflammation by mediating NF-κB phosphorylation and NLRP3/ASC/caspase-1 activation.

### GO blocks the expression of inflammatory pyroptosis-related proteins in LPS-induced ALI

**Figure 4:**
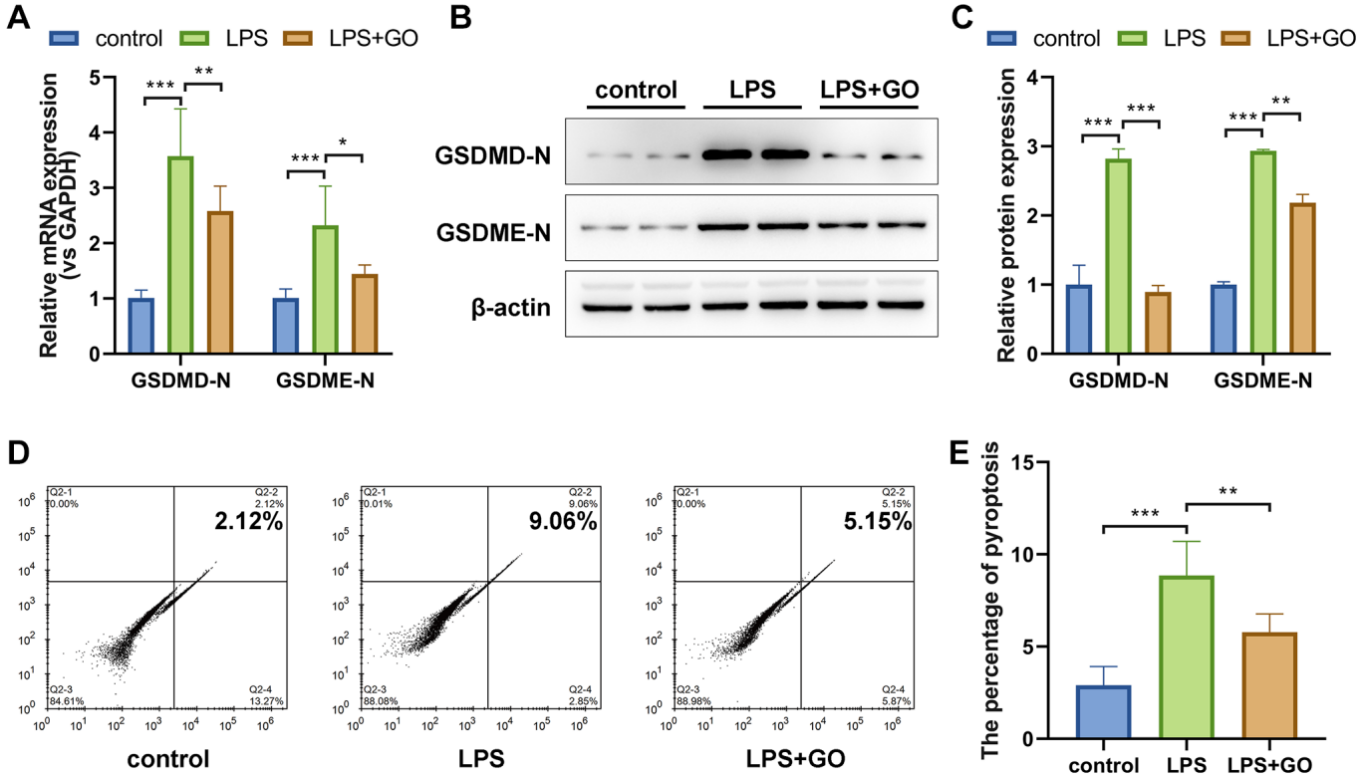
**GO blocks the expression of pyroptosis-related proteins in LPS-induced ALI.** (**A**) mRNA expression levels of the active forms of the pyroptosis-related proteins GSDMD and GSDME in lung tissue were analyzed via real-time PCR. (**B**) Protein expression levels of the active forms of the pyroptosis-related proteins GSDMD and GSDME in lung tissue were analyzed via western blotting. (**C**) A histogram of the grayscale values of the protein bands calculated using ImageJ software (standardized to β-actin). (**D**) The pyroptosis rate of isolated primary alveolar epithelial cells was analyzed using flow cytometry and FLICA caspase-1 and PI double staining. (**E**) The incident rate of pyroptosis was calculated for each group. Experiments were performed in triplicate, and data are expressed as the mean ± standard deviation (SD). Student’s *t*-test, ^*^p < 0.05, ^**^p < 0.01, and ^***^p < 0.001. Abbreviation: ns: no significant difference.

Both the activation of the NLRP3/ASC/caspase-1 inflammasome complex and the substantial release of inflammatory cytokines, especially IL-1β and IL-18, in damaged lungs indicated that LPS-induced ALI may be associated with inflammatory pyroptosis. Hence, we conducted real-time PCR and western blotting to measure the expression levels of recognized pyroptosis marker proteins in the lung, the gasdermin family (GSDME and GSDMD). The active form of gasdermins was significantly upregulated in LPS-induced ALI, which was effectively reversed by GO pretreatment (Figure 4A–4C). Subsequently, we investigated the pyroptosis ratio of primary alveolar epithelial cells via flow cytometry using propidium iodide and FLICA660 YVAD double staining. The proportion of pyroptotic cells was remarkably increased in LPS-induced ALI (from 2.12% to 9.06%) compared with control cells but was further reduced in GO-pretreated cells (from 9.06% to 5.15%) (Figure 4D, 4E). Collectively, these findings indicate that GO may alleviate LPS-induced ALI by blocking inflammation-related pyroptosis.

### GO attenuates LPS-induced ALI via H_2_S generation

**Figure 5:**
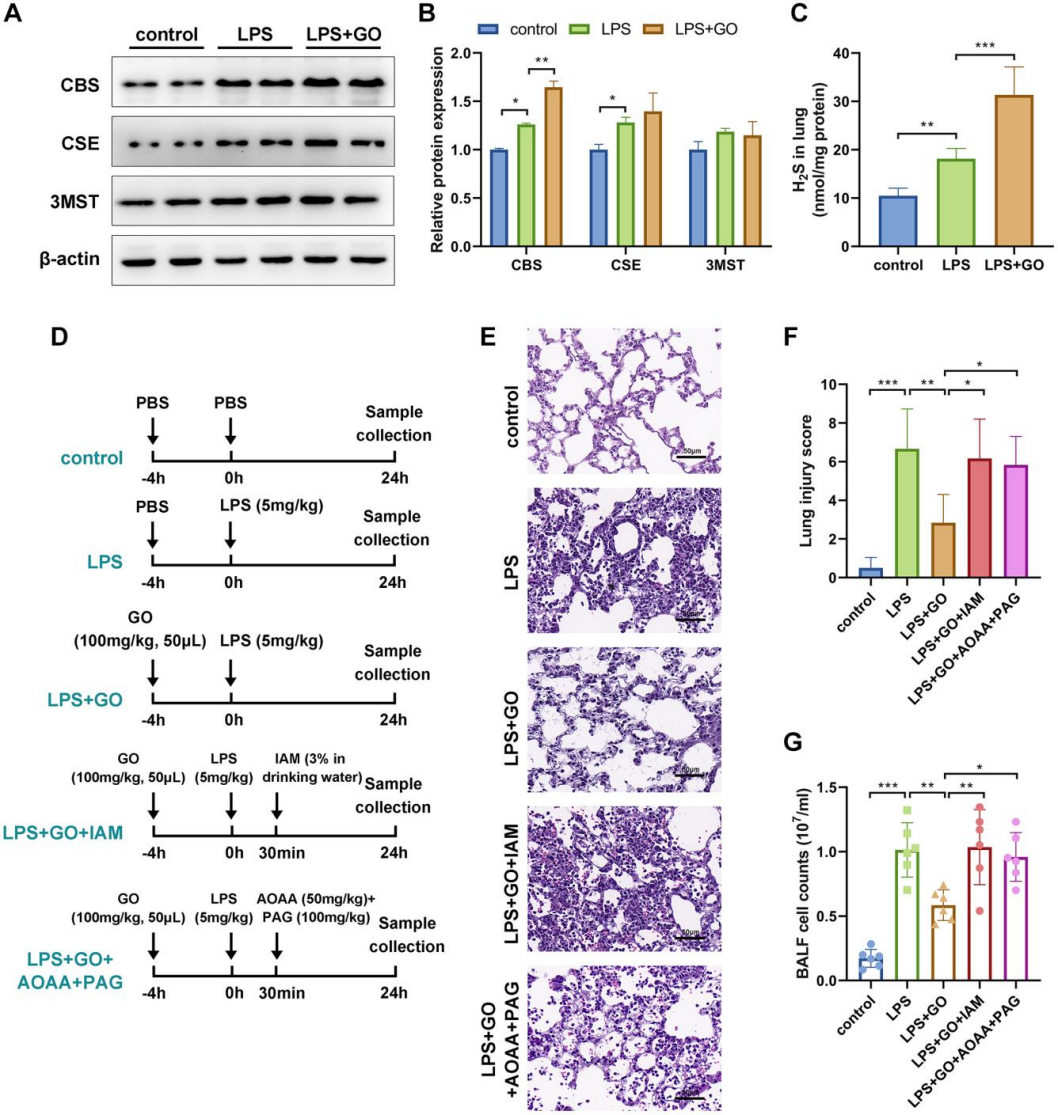
**GO alleviated LPS-induced ALI via the H_2_S generating pathway.** (**A**) Western blotting to detect the protein expression levels of three H_2_S-producing enzymes, namely CBS, CSE, and 3MST, in lung tissue. (**B**) The grayscale values of the bands in image A were normalized to β-actin and analyzed using ImageJ software. (**C**) Detection of H_2_S levels in lung tissues. (**D**) Flow diagrams of mice experiments. IAM is an effective inhibitor of the conversion of DATS to H_2_S *in vivo*. For IAM delivery, mice were administered 3% IAM in their drinking water 30 min after LPS instillation. AOAA is a CBS inhibitor and PAG is a CSE inhibitor. For AOAA and PAG delivery, mice were intraperitoneally injected with AOAA (50 mg/kg) and PAG (100 mg/kg), respectively, 30 min after LPS instillation. (**E**) Lung tissue sections stained with hematoxylin and eosin (H&E staining, scale bar: 50 μm). (**F**) Severity of lung damage was scored on a scale of 0–10 according to inflammatory cell infiltration, alveolar septal edema and thickening, hyaline membrane formation, and pulmonary hemorrhage (*n* = 8 per group). (**G**) The total number of cells exuded in BALF. Data are expressed as the mean ± SD. Student’s *t*-test, ^*^p < 0.05, ^**^p < 0.01, and defined as ^***^p < 0.001. Abbreviation: ns: no significant difference.

Garlic is rich in polysulfide compounds, including DATS and diallyl disulfide, which liberate H_2_S *in vitro*. Additionally, GO therapy significantly increases the expression of H_2_S-producing enzymes, including CBS, CSE, and 3MST, in renal tissue [32]. H_2_S reduces pyroptosis and alleviates acute kidney or lung injury by suppressing the NLRP3 inflammasome [36, 44]. Therefore, we further investigated whether the protective effects of GO on LPS-induced ALI were due to the release of H_2_S. First, we detected the effects of GO on the expression of three H_2_S-producing enzymes, including CBS, CSE, and 3MST, in lung tissue via western blotting. The results revealed that the LPS-treated group displayed a slight increase in the expression levels of CBS and CSE compared with the controls, whereas a remarkable increase in the expression levels of CBS and CSE was observed in the GO pretreatment group. However, the expression levels of 3MST did not differ among the three groups (Figure 5A, 5B). Consistent with the above results, we found that LPS treatment slightly increased H_2_S production, whereas GO pretreatment substantially increased H_2_S production in lung tissue compared with the control group (Figure 5C).

Based on our observation that GO treatment significantly increases H_2_S production, we hypothesized that the protective effects of GO in LPS-induced ALI are associated with H_2_S production. To confirm this hypothesis, we continued to investigate whether the removal of H_2_S could reverse the protective effect of GO. H_2_S in the lungs can be converted by polysulfide compounds in garlic, such as DATS (exogenously produced H_2_S) or can be produced *in vivo* by H_2_S-producing enzymes (endogenously produced H_2_S). Therefore, IAM (an effective inhibitor of the conversion of DATS to H_2_S *in vivo*) was used to remove exogenous H_2_S, and AOAA (CBS inhibitor) and PAG (CSE inhibitor) were used to remove endogenous H_2_S (Figure 5D). The lung histological pathology revealed that GO pretreatment significantly improved LPS-induced lung injury, whereas cotreatment with IAM or AOAA and PAG reversed the protective effects of GO on ALI and increased the lung injury scores again (Figure 5E, 5F). The results from cell counts in BALF revealed a similar trend (Figure 5G). These results indicate that GO attenuated LPS-induced ALI via the H_2_S-generating pathway.

### GO inhibits NF-κB and NLRP3 inflammasome-mediated inflammatory pyroptosis in LPS-induced ALI via H_2_S generation

**Figure 6:**
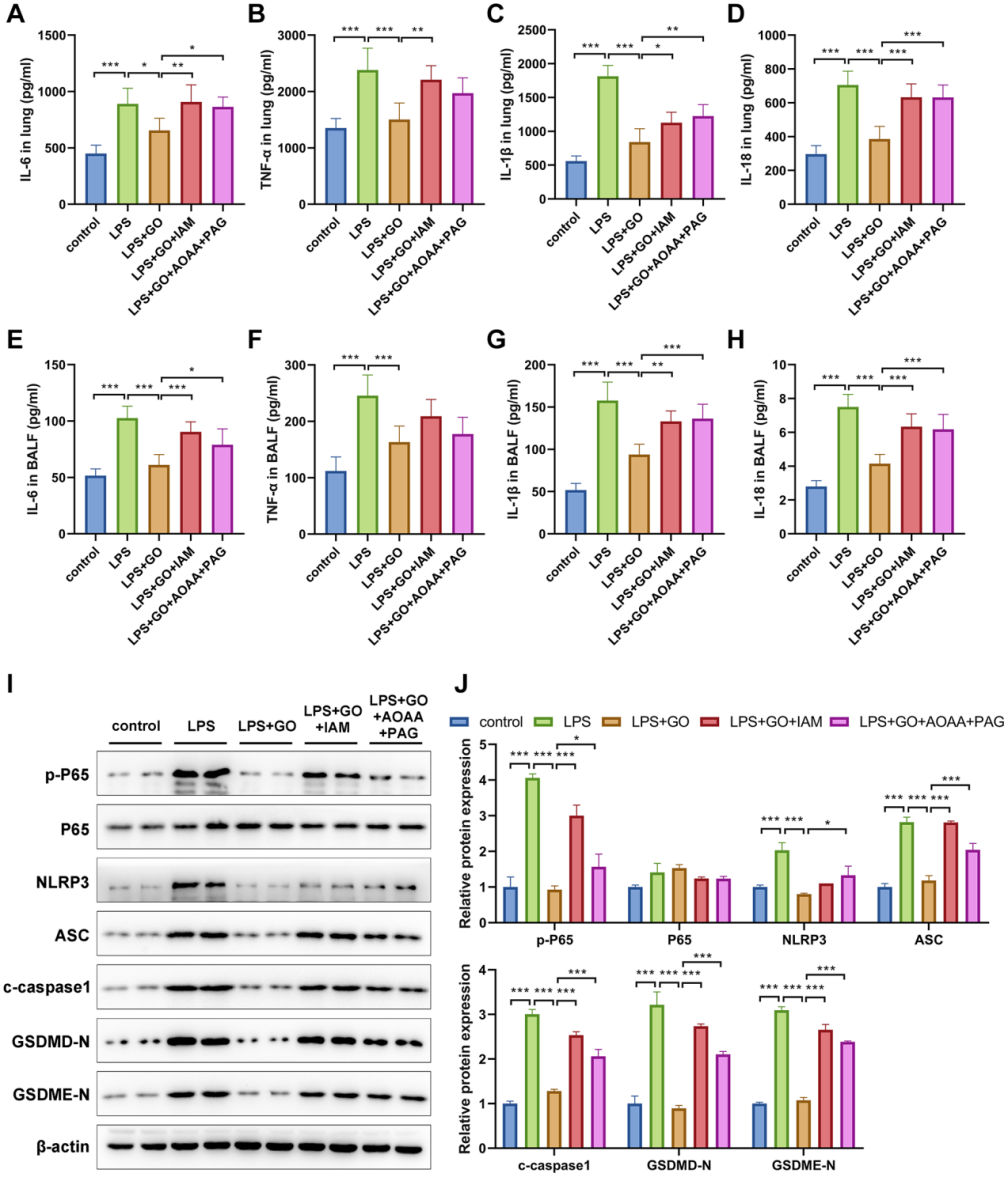
**GO inhibits the NF-κB signaling and NLRP3 inflammasome-mediated inflammatory pyroptosis in LPS-induced ALI via the H_2_S-generating pathway.** (**A**–**D**) ELISA of the levels of inflammatory cytokines, including TNF-α, IL-6, IL-1β, and IL-18, in lungs. (**E**–**H**) ELISA of the levels of secreted inflammatory cytokines, including TNF-α, IL-6, IL-1β, and IL-18, in BALF. (**I**) Western blotting to detect the protein expression levels of p-p65, p65, NLRP3, ASC, c-caspase 1, and apoptotic markers in lungs. (**J**) The grayscale values of the bands in the image were normalized to β-actin and analyzed using ImageJ software. Data are expressed as the means ± SD. Student’s *t*-test, ^*^p < 0.05, ^**^p < 0.01, and ^***^p < 0.001. Abbreviation: ns: no significant difference.

Next, we explored whether the potential mechanism underlying the suppressive effects of GO on NF-κB and NLRP3 inflammasome-associated inflammatory pyroptosis was related to the production of H_2_S. First, we measured the levels of the inflammatory cytokines TNF-α, IL-6, IL-1β, and IL-18 in the lungs and BALF of ALI mice in different groups via ELISA. The results revealed that GO inhibited the expression of inflammatory cytokines in the lungs and BALF in LPS-induced ALI. However, the inhibitory effects of GO on inflammatory cytokine expression were significantly attenuated upon treatment with IAM or a combination of AOAA and PAG (Figure 6A–6H). Additionally, we observed that GO effectively inhibited the expression of p-p65/p65, NLRP3, ASC, cleaved caspase-1, GSDMD-N, and GSDME-N in LPS-induced ALI. However, when cotreated with IAM or AOAA and PAG to remove exogenous or endogenous H_2_S, the inhibitory effects of GO on the activation of NF-κB, NLRP3 inflammasome complex, and pyroptosis in LPS-induced ALI were significantly weakened (Figure 6I, 6J). Accordingly, we propose that GO inhibited NF-κB and NLRP3 inflammasome-mediated inflammatory pyroptosis in LPS-induced ALI and that this phenomenon was partially associated with the H_2_S-generating pathway.

## DISCUSSION

ALI is a significant contributor to critical acute respiratory failure [45]. To date, therapeutic strategies specific to ALI remain limited. With the continuous development of modern medicine, many active ingredients from plants or food with antimicrobial, anti-inflammatory, and antioxidant activities have been identified to exhibit some degree of efficacy in preventing and treating ALIs [18, 19]. Garlic, a globally used cooking spice, has numerous medicinal effects. It has anti-inflammatory, immunomodulatory, and metabolic regulatory properties, playing a preventive and protective role in various degenerative illnesses, such as hyperlipidemia, cardiovascular disease, and cancer [20, 21, 32, 46]. This study aimed to explore the therapeutic effect of GO on LPS-induced ALI. Rewardingly, we discovered that GO pretreatment significantly inhibited LPS-induced ALI, including improving lung pathology, reducing the lung W/D ratio, and decreasing inflammatory cell counts and protein levels in BALF samples.

To date, the pathogenesis of ALI remains unclear. Immunomodulatory and inflammatory processes play a non-negligible role in the progression of multifactorial-promoted ALIs [47]. Inflammation, in particular, is recognized as a major contributor to lung injury [48]. An inflammatory response consisting of a complex network of cytokines has been found to be a major feature of LPS-induced ALI [49]. Hence, we explored the function of GO in regulating inflammatory responses in LPS-induced ALI. Consistent with the above study, we found elevated levels of inflammatory cytokines, such as TNF-α, IL-6, IL-1β, and IL-18, in the lungs and BALF samples of mice with LPS-induced ALI. However, the upregulation of inflammatory cytokine levels in the lungs and BALF samples in ALI was prevented by GO pretreatment. Additionally, studies have revealed that the severity of ALI may be exacerbated if pulmonary neutrophil infiltration occurs during the early stages of the inflammatory processes of ALI [50–52]. The activity of MPO, an enzyme contained within neutrophils, is a useful indicator of pulmonary infiltration of neutrophils. In this study, we also found that the LPS-induced upregulation of MPO activity was significantly inhibited by GO. These results strongly indicate that GO effectively protected the lung from LPS-induced inflammation injury.

Among the various inflammatory pathways, the NF-κB pathway and NLRP3 inflammasome are the primary molecular pathways. NF-κB has been reported as a key signaling molecule with an important role in regulating inflammatory mediators [53]. NF-κB overresponse worsens lung inflammation [43]. The NLRP3 inflammasome, which is activated by the assembly of the three components NLRP3, ASC, and caspase-1 into a protein complex, is a prerequisite for the maturation and release of IL-1β and IL-18 [54]. Suppression of NLRP3 activation alleviates LPS-induced ALI [13]. We explored GO regulation of inflammation by targeting the NF-κB pathway and NLRP3 inflammasome. Our results revealed that GO remarkably inhibited the LPS-induced activation of NF-κB and NLRP3 inflammasome. Pyroptosis is an emerging form of inflammatory cell death that frequently occurs during the pathogenesis of inflammation, and the NLRP3 inflammasome contributes to the induction of pyroptosis in LPS-induced ALI [55]. In the present study, we confirmed that pyroptosis occurred and that the levels of pyroptosis markers, such as GSDMD and GSDME, were elevated in LPS-induced ALI but were reduced by GO pretreatment. Our data suggested that GO inhibited LPS-induced ALI by suppressing the NF-κB pathway and NLRP3 inflammasome as well as inflammatory-related pyroptosis.

Many organic polysulfides, such as DATS and diallyl disulfide, are present in GO and release H_2_S under physiological conditions. Thus, GO is an exogenous H_2_S donor. Additionally, other studies and our experimental results both demonstrated that GO could significantly increase the expression level of H_2_S-producing enzymes *in vivo*, thereby increasing the production of endogenous H_2_S [32]. Recently, an increasing number of studies have revealed that H_2_S is not only a toxic gas molecule but also has many beneficial physiological effects, such as antiapoptosis, antioxidation, and anti-inflammation. H_2_S can also prevent acute endotoxemia-induced lung injury [28, 29]. Accordingly, we aimed to explore whether the protective effect of GO on LPS-induced ALI was due to the H_2_S-generating pathway. We found that the protective effects of GO on ALI as well as the inhibitory effects of GO on the NF-κB pathway, NLRP3 inflammasome, and inflammatory pyroptosis were reversed when endogenous and exogenous H_2_S were removed. Our findings indicated that H_2_S production may be the major mechanism underlying the protective effect of GO on LPS-induced ALI.

In conclusion, our findings suggested that GO had a protective effect on LPS-induced ALI in mice via the suppression of the inflammatory response. The inhibitory effects were due to the inhibition of NF-κB, NLRP3, and the inflammatory pyroptotic pathway. GO exerts these effects by regulating the H_2_S-generating pathway. In summary, our study demonstrates that the application of GO helps prevent LPS-induced ALI. In a future study, positive control and biosafety analyses will be performed to further explore the clinical potential of GO.
